# Lack of impact of nil-per-os (NPO) time on goal-directed fluid delivery in first case versus afternoon case starts: a retrospective cohort study

**DOI:** 10.1186/s12871-019-0864-x

**Published:** 2019-10-27

**Authors:** R. Ryan Field, Tuan Mai, Samouel Hanna, Brian Harrington, Michael-David Calderon, Joseph Rinehart

**Affiliations:** 1 Department of Anesthesiology & Perioperative Care, University of California Irvine, 101 The City Drive South, Orange, CA 92868 USA; 2 Wake Forest School of Medicine, Winston-Salem, USA

**Keywords:** Fluid Management, Goal Directed Fluid Management, Goal Directed Fluid Therapy, NPO

## Abstract

**Background:**

Goal Directed Fluid Therapy (GDFT) represents an objective fluid replacement algorithm. The effect of provider variability remains a confounder. Overhydration worsens perioperative morbidity and mortality; therefore, the impact of the calculated NPO deficit prior to the operating room may reach harm.

**Methods:**

A retrospective single-institution study analyzed patients at UC Irvine Medical Center main operating rooms from September 1, 2013 through September 1, 2015 receiving GDFT. The primary study question asked if GDFT suggested different fluid delivery after different NPO periods, while reducing inter-provider variability. We created two patient groups distinguished by 0715 surgical start time or start time after 1200. We analyzed fluid administration totals with either a 1:1 crystalloid to colloid ratio or a 3:1 ratio. We performed direct group-wise testing on total administered volume expressed as total ml, total ml/hr., and total ml/kg/hr. between the first case start (AM) and afternoon case (PM) groups. A linear regression model included all baseline covariates that differed between groups as well as plausible confounding factors for differing fluid needs. Finally, we combined all patients from both groups, and created NPO time to total administered fluid scatterplots to assess the effect of patient-reported NPO time on fluid administration.

**Results:**

Whether reported by total administered volume or net fluid volume, and whether we expressed the sum as ml, ml/hr., or ml/kg/hr., the AM group received more fluid on average than the PM group in all cases. In the general linear models, for all significant independent variables evaluated, AM vs PM case start did not reach significance in both cases at *p* = 0.64 and *p* = 0.19, respectively. In scatterplots of NPO time to fluid volumes, absolute adjusted and unadjusted R2 values are < 0.01 for each plot, indicating virtually non-existent correlations between uncorrected NPO time and fluid volumes measured.

**Conclusions:**

This study showed NPO periods do not influence a patient’s volume status just prior to presentation to the operating room for surgical intervention. We hope this data will influence the practice of providers routinely replacing calculated NPO period volume deficit; particularly with those presenting with later surgical case start times.

## 1. Key points

**Question:** Does nil-per-os (NPO) time and preoperative fasting influence fluid needs during surgery?

**Findings:** Using multiple statistical analyses, we found no relationship between NPO time and fluid volume delivery suggested by a goal-directed fluid-therapy algorithm.

**Meaning:** Intravenous fluid resuscitation based on historical “NPO time deficits” may not be indicated.

## 2. Introduction

A central and still somewhat controversial question in anesthetic care asks whether nil-per-os (NPO) period fluid-deficits need intraoperative replacement [1]. Previous work suggested the NPO period may not require replacement; however, these studies were often investigated with invasive measures and failed to eliminate the potential for provider-based differences in the fluid delivery schema [2–4]. When considering how intraoperative over-hydration worsens perioperative outcomes, including morbidity and mortality, the specifics of the calculated volume of NPO fluid deficit ‘replaced’ becomes more important [5–9]. A systematic review by the International Fluid Optimization Group of 162 different papers on fluid delivery in different surgical patient populations revealed decreased hospital length of stay, less postoperative complications, earlier recovery of gut function, and reduced need for intensive care unit (ICU) therapy in most patients when treated with goal directed fluid therapy (GDFT) [4].

GDFT stands as a validated and objective fluid replacement algorithm that also significantly reduces the impact of provider-related variability in fluid delivery [4, 5, 8, 10–13, 15]. Thus, a straightforward way to test the effect of the NPO period on intraoperative fluid requirements would review the fluid delivery delivered by an objective algorithm in first case start (AM) versus afternoon case start (PM) with substantially longer NPO time in the latter group. We set out to retrospectively analyze whether or not GDFT algorithm suggested different fluid delivery after different NPO periods. Our null hypothesis supposed the longer NPO period for PM surgical case starts would increase the total fluid recommended by the goal-directed fluid therapy algorithm.

## 3. Methods

This retrospective study utilized a de-identified dataset provided by the hospital information technology department. The University of California Irvine Institutional Review Board deemed the protocol IRB exempt.

### 3.1. Data collection

We extracted data for this study from our perioperative database, SIS (Surgical Information Systems, Alpharetta, GA). The initial data pull included all adult procedures in 20 main operating rooms at UC Irvine Medical Center (UCIMC) from September 1, 2013 through September 1, 2015 that were marked as receiving GDFT in the medical record (our charts include a mandatory GDFT field that must be selected yes/no before the chart can be closed).

Inclusion criteria for the study were adult patients age 18 or over having open or laparoscopic abdominal procedures (colectomy, adrenalectomy, gastrectomy, hepatic resection, Whipple or pancreatic procedures, nephrectomy, cystectomy, abdominoperineal resection, or gynecologic oncology procedures). For each identified case, we pulled: case, date and time; procedure; patient demographics (including gender, height, weight, age, American Society of Anesthesiologists Patient Score); patient comorbidities (including hypertension, congestive heart failure, renal failure, and dialysis); NPO time; whether or not the patient received an epidural or arterial line; intraoperative data (including urine output, estimated blood loss, total crystalloid and colloid, blood administration, median and minimum heart rate, median and minimum mean arterial pressure).

We sorted the data and created two groups distinguished as either AM (0715 surgical start time) or PM (after 1200 surgical start time). Cases starting between 0730 and 1200 were excluded to ensure distinct separation between the two groups. Following this, we applied further exclusions to standardize the patient cohorts and reduce variability due to surgical factors: patients less than 18 or older than 100 years; patients with estimated blood loss (EBL) greater than 500 mL or who received blood products intraoperatively; and those with congestive heart failure (CHF), end-stage renal disease (ESRD), or who were receiving dialysis. We also excluded emergency cases, patients admitted for greater than 24 h prior to surgery (since intravenous fluid could have been administered during the NPO period), and those who received hypertonic bowel preparations.

### 3.2. Institutional NPO protocols

We instructed all patients to fast at midnight before surgery. If patients needed any PO medications prior to surgery, we instructed them to take these with a sip of water. Patients arrived in the preoperative holding unit roughly 2 h prior to the start of surgery. Preoperative nurses obtained intravenous access and started crystalloids (normal saline or Ringer’s lactate) at keep vein open (KVO) flows.

### 3.3. Goal-directed fluid therapy protocol

**Fig. 1:**
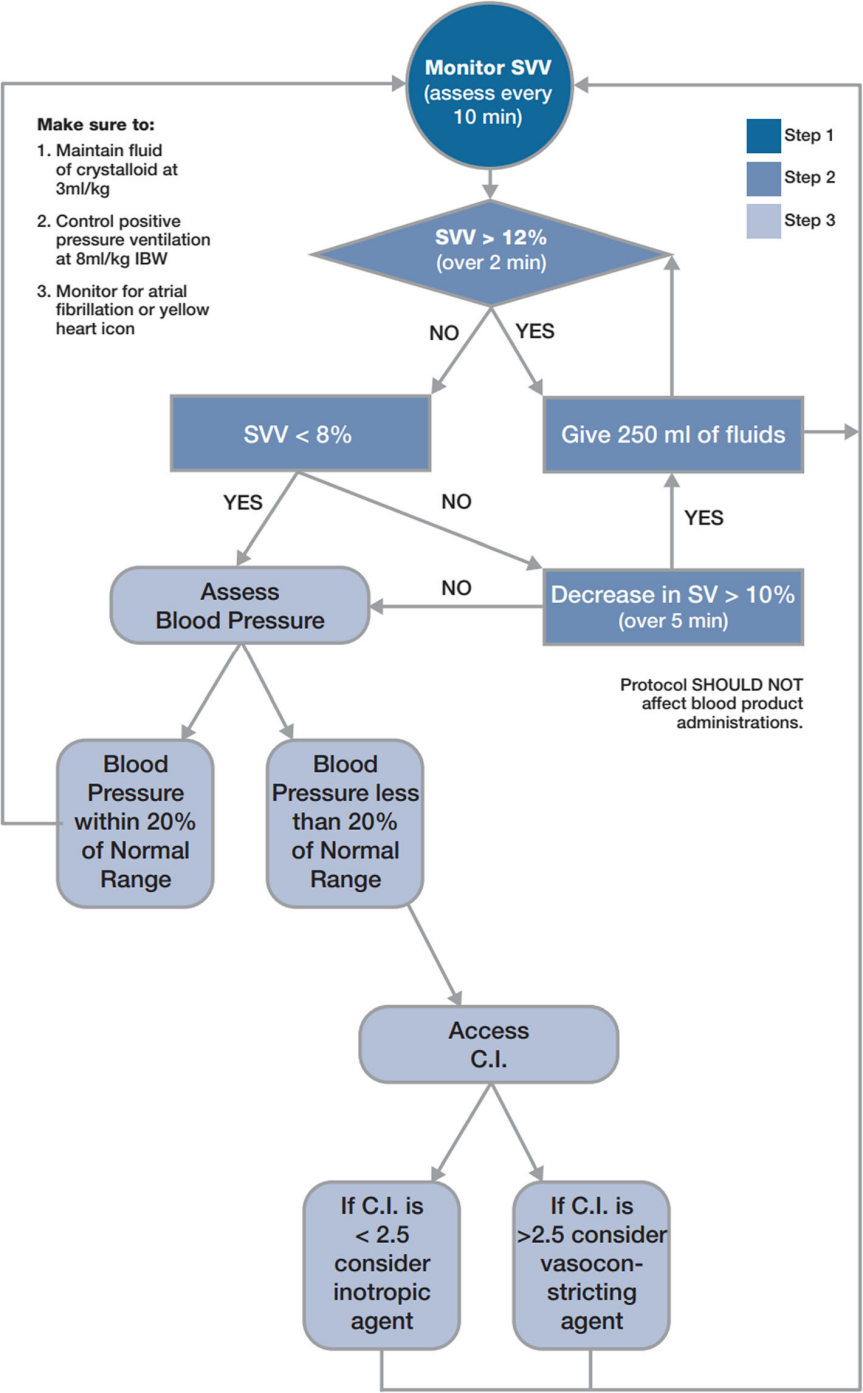
Goal-directed fluid therapy protocol in use at UCI Medical Center during the study period. CI – Cardiac index. IBW – ideal body weight. SV – stroke volume. SVV – stroke volume variation

The primary GDFT protocol in use at UCIMC during this time period was an adaptation of the stroke volume variation (SVV) protocol and is outlined in Fig. 1. Additional protocol information is available in the appended GDFT Protocol and GDFT Overview (Additional files 1 and 2) We monitored patients who did not have arterial lines either by transesophageal Doppler, non-invasive continuous blood pressure monitoring, or by plethysmograph variability index (Masimo corp, Irvine, CA). Our database unfortunately did not record specific monitoring device.

### 3.4. Statistical analysis & outcomes

Primarily we sought to determine whether PM patients required different fluid delivery when compared to AM patients when a GDFT protocol recommended care. Our statistical approach therefore assessed this question from many possible perspectives to determine whether any evidence exists to support different fluid delivery requirements in AM compared to during PM surgery. We calculated and analyzed fluid administration totals with both a 1:1 crystalloid to colloid ratio as well as with a 3:1 crystalloid to colloid ratio.

First, direct group-wise testing was performed on total administered volume (crystalloid + colloid) calculated as total ml, total ml/hr., and total ml/kg/hr. between the AM and PM groups. These three different calculations allowed us to check raw total, raw total corrected for duration of case, and raw total corrected for duration of case and size of patient. Second, a linear regression model was run that included all of the baseline covariates that differed between groups as well as the plausible confounding factors for differing fluid needs (ASA class, use of epidural anesthesia, laparoscopic vs. open case). Our model used a 1:1 crystalloid to colloid ratio or a 3:1 crystalloid to colloid ratio, which allowed us to evaluate the marginal influence of group (AM or PM) in light of all of the other covariates. Finally, we combined all patients from both groups and scatterplots of NPO time to total fluid (as ml, ml/hr., and ml/kg/hr) assessed the effect of patient-reported NPO time on fluid administration.

We assumed group size imbalance may exist between AM and PM case starts due to the consistent morning starts in all ORs. We knew if we could pull at least 300 patients into the former and 100 patients into the latter, assuming the typical patient received 2100 ± 450 ml of fluid based on previous work [12], with a power of 0.8 and alpha of 0.05, we would sufficiently power our analysis to detect a difference of approximately 300 ml of fluid required between the groups. With a minimum reported NPO time difference of 2.5 h between groups, this represented the ability to detect a need of as little as 120 ml of ‘deficit’ fluids between groups per hour of NPO time.

We performed statistical analyses using SPSS (IBM, Armonk NY) or R (http://www.R-project.org). We report data as mean ± standard deviation, or as count (percentile) for categorical variables. Because we assessed *any* possible increase in need for fluid, we considered a *p*-value of < 0.05 significant with no corrections for multiple comparisons made.

## 4. Results

**Fig. 2:**
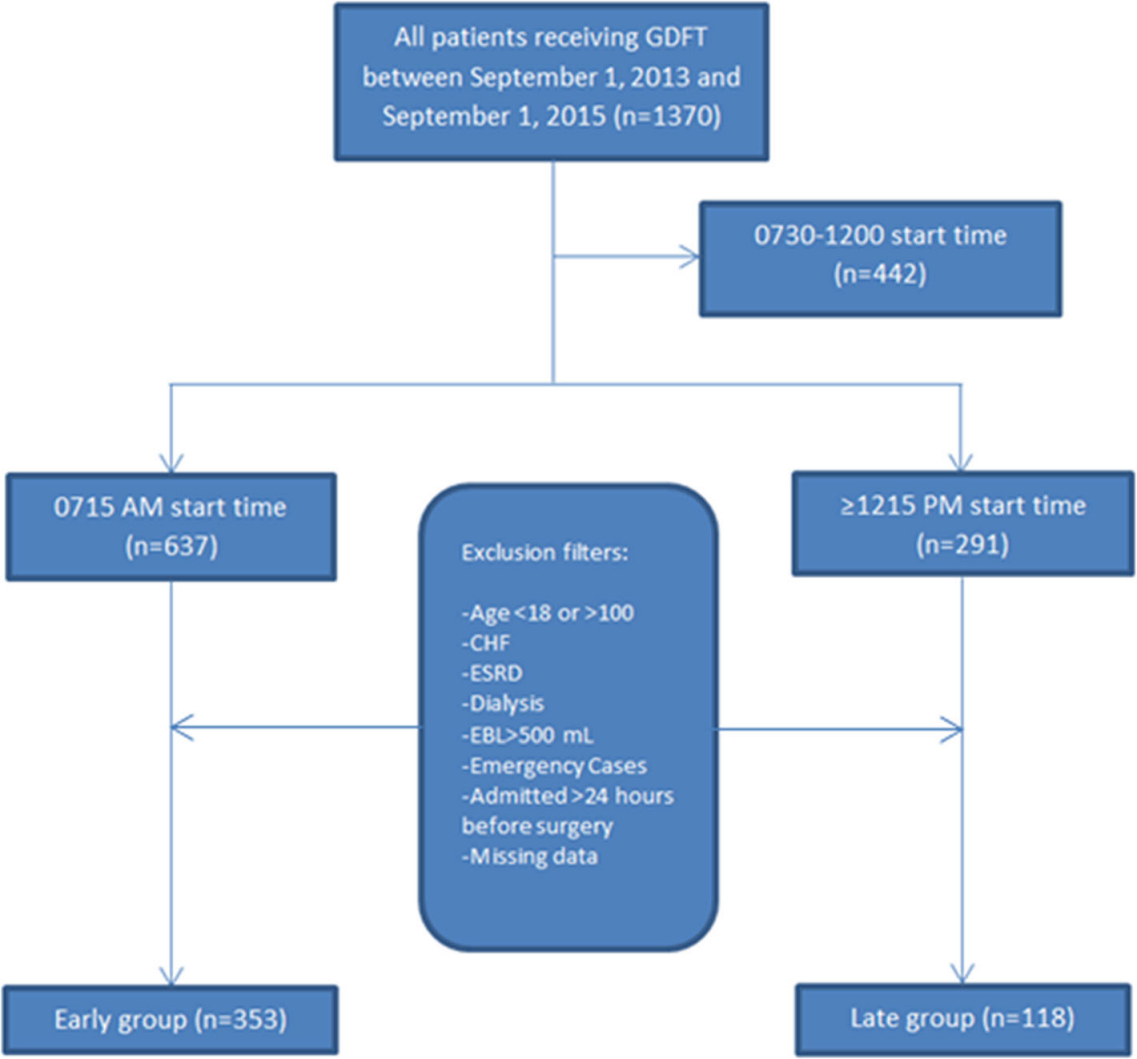
Patient Selection and Group Allocation. GDFT – Goal Directed Fluid Therapy; CHF – congestive heart failure; ESRD – end-stage renal disease; EBL – estimated blood loss

Our initial search pulled 1370 patients that we flagged as receiving GDFT during the study period. After filtering out patients with the study inclusion and exclusion criteria as detailed in Fig. 2, a total of 471 patients met criteria within the two-year study period that we then used in data analysis, 353 in the AM group and 118 in the PM group.

Table 1 details baseline demographics for each group. Patient ages, genders, heights, weights, ASA classifications, and EBL did not significantly differ between groups. Table 2 describes the distribution of GDFT monitoring modalities. We observed a significantly higher rate of epidural use, arterial line placement, and longer case duration in the AM group compared to the PM group (*p* < 0.001 for all comparisons). AM case starts also saw a higher proportion of laparoscopic cases than PM cases (37% vs. 23%, *p* = 0.005). Finally, mean NPO time was shorter in the AM group than the PM group (10.0 ± 2.5 h vs. 12.5 ± 3.1 h (median and interquartile ranges 10 [8–12] and 12 [10–12] respectively), a statistically significant difference at *p* < 0.001). Actual NPO distribution data can be found appended.

**Table 1:** Demographic and Baseline Data in Both Groups

Variable	AM (*n* = 353)	PM (*n* = 118)	*p*-value for group difference
Age (years)	60 ± 14	60 ± 17	0.9
Gender			
Male (%)	166 (47%)	53 (45%)	0.75
Female (%)	187 (53%)	65 (55%)	
Height (cm)	169 ± 11	168 ± 11	0.38
Weight (kg)	78 ± 18	77 ± 21	0.74
BMI (kg/m^2)	27.1 ± 5.5	27 ± 6	
ASA Class			
I	3 (0.9%)	1 (0.9%)	0.19
II	63 (18%)	27 (23%)	
III	249 (71%)	76 (64%)	
IV	38 (11%)	15 (12%)	
Procedure			
Laparoscopic	131 (37%)	27 (23%)	**0.005**
Open	222 (63%)	91 (77%)	
Epidural	105 (30%)	14 (12%)	**< 0.001**
Arterial Line	271 (77%)	55 (47%)	**< 0.001**
NPO time (hours)	10.0 ± 2.5	12.5 ± 3.1	**< 0.001**
Duration (hours)	6.2 ± 2.7	4.1 ± 1.9	**< 0.001**
EBL (milliliters)	129 ± 135	107 ± 128	0.12

Two-sample t-test for continuous variables and Pearson's chi-squared test for binary/categorical variables. *ASA* American Society of Anesthesiologists, *BMI* Body Mass Index, *NPO* nil per os. P-values bolded of variables with significant difference between the AM vs PM groups

**Table 2:** GDFT Monitoring Modality

GDFT Monitoring Modality	Percent Utilization (%)
Esophageal Doppler	4
Plethysmograph PVI	30
Arterial Line PPV	41
Non-invasive Continuous Finger Cuff	25

Table 3 summarizes fluid totals in both groups as calculated by the different approaches (‘Net’ vs. ‘Administered’ and 1:1 vs. 3:1). Whether summarized by total administered volume or net fluid volume, and whether the sum was expressed as ml, ml/hr., or ml/kg/hr., the AM group received *more* fluid on average than the PM group in all cases. In half of the approaches to calculating fluid totals the higher AM case total was statistically significant; in the other half the difference showed was not. However, no approach to calculating fluid delivery revealed a recommendation by the GDFT algorithm to administer more total fluid volume in PM cases than any AM cases.

**Table 3:** Administered Fluid and Net Fluid Balances between groups

Total Expressed As	Crystalloid to Colloid Calculation	Volume expressed as	AM Cohort	PM Cohort	*p*-value
Administered Volume	1:1	Total ml	1970 ± 1220	1300 ± 850	**< 0.001**
		ml/hr	340 ± 180	300 ± 160	**0.04**
		ml/kg/hr	4.5 ± 2.5	4.0 ± 2.3	0.07
	1:3	Total ml	1000 ± 690	640 ± 490	**< 0.001**
		ml/hr	164 ± 91	145 ± 92	0.07
		ml/kg/hr	2.2 ± 1.3	2.0 ± 1.3	0.08
Net Fluid Balance (volume)	1:1	Total ml	1230 ± 1120	800 ± 810	**< 0.001**
		ml/hr	220 ± 180	190 ± 170	0.16
		ml/kg/hr	2.9 ± 2.5	2.5 ± 2.3	0.17
	1:3	Total ml	270 ± 660	140 ± 500	**0.05**
		ml/hr	46 ± 103	36 ± 108	0.42
		ml/kg/hr	0.6 ± 1.4	0.4 ± 1.4	0.35

Bold data denotes *P*-values less than 0.05

The general linear models, seen in Tables 4 and 5, with start time groups (AM vs PM) including all unmatched baseline variables, in the first columns, and other plausible drivers of fluid administration, whether fluid was totaled as 1:1 or 3:1 crystalloid to colloid, showed significant independent variables to be ASA classification, placement of an epidural catheter, urine output, estimated blood loss, patient weight, patient age, and surgical duration. AM vs. PM case start time did not significantly differ in either group at *p* = 0.64 and *p* = 0.19, respectively. When raw reported NPO is used as the duration of NPO for each patient group instead of AM vs. PM groupings in the models, NPO time is also non-significant as an independent variable at *p* = 0.38 and *p* = 0.97 in the 1:1 and 3:1 fluid total models, respectively.

**Table 4:** Linear Model Results with AM vs PM Start Time

Variable	*p*-value in general linear model	
	1:1 Crystalloid:Colloid	0.33:1 Crystalloid:Colloid
Intercept	0.785	0.360
Group (AM vs. PM)	0.640	0.187
History of Hypertension	0.380	0.209
Gender	0.476	0.081
ASA	0.018	0.002
Epidural Placed	0.002	0.001
Arterial Line Used	0.586	0.861
Laparoscopic	0.215	0.160
Urine Output	0.022	0.004
Estimated Blood Loss	0.000	0.000
Patient Weight	0.000	0.009
Patient Age	0.004	0.016
Sugical Duration	0.001	0.000
Median MAP	0.571	0.242
Median HR	0.090	0.108

**Table 5:** Linear Model Results with NPO Time

Variable	p-value in general linear model	
	1:1 Crystalloid:Colloid	0.33:1 Crystalloid:Colloid
Intercept	0.294	0.170
NPO time (hours)	0.382	0.971
History of Hypertension	0.660	0.516
Gender	0.675	0.450
ASA	0.004	0.010
Laparoscopic	0.001	0.001
Epidural Placed	0.001	0.000
Arterial Line Used	0.582	0.075
Urine Output	0.002	0.000
Estimated Blood Loss	0.001	0.000
Patient Weight	0.001	0.016
Patient Age	0.082	0.044
Sugical Duration	0.000	0.000
Median MAP	0.662	0.185
Median HR	0.006	0.011

**Fig. 3:**
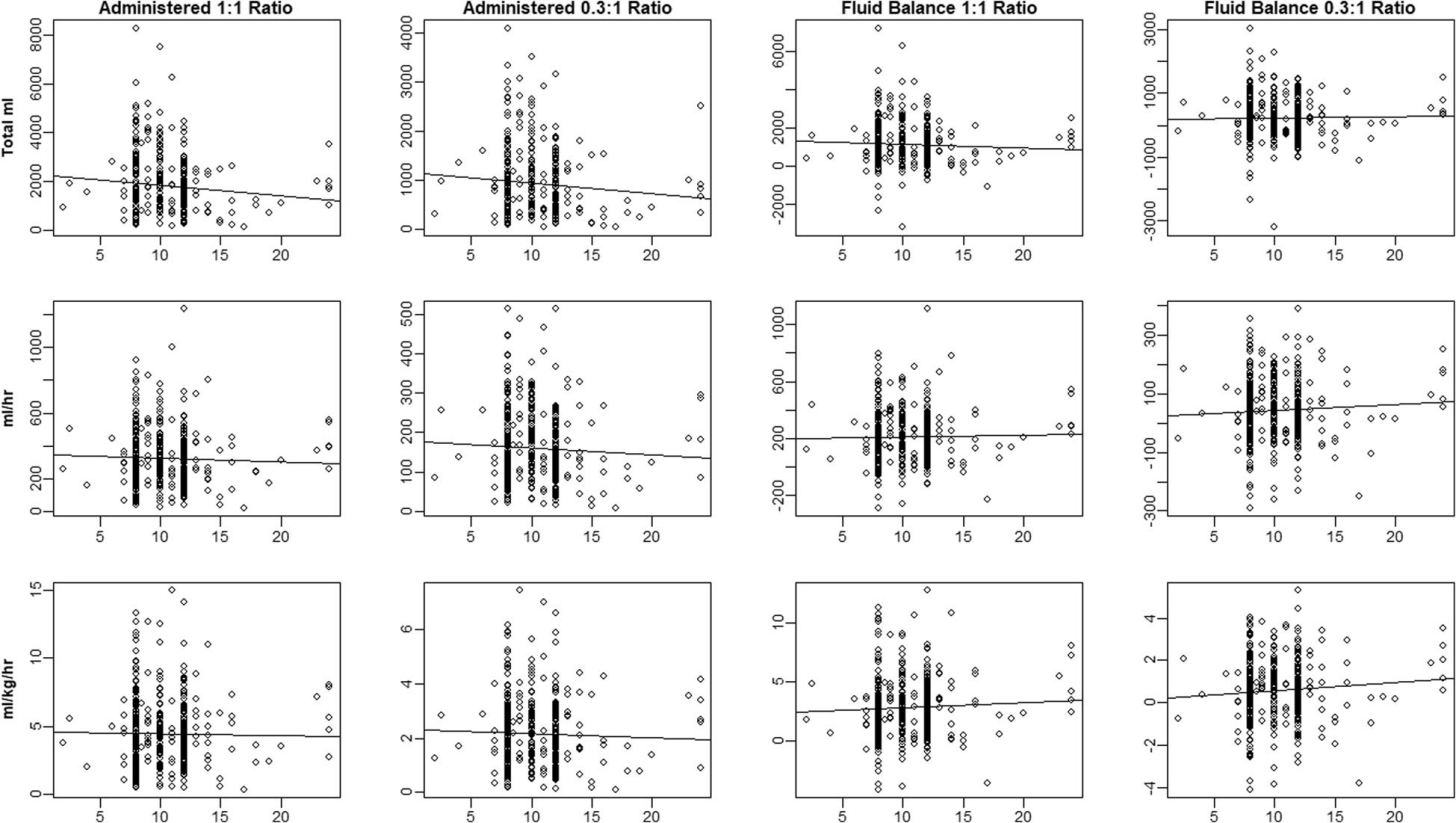
NPO Time Compared to Fluid Requirements by Goal-Directed Fluid Therapy. Regression lines for twelve different approaches to fluid-administration estimation. Each row represents a different calculation of amount administered: top row – total ml; middle row – ml/hour; bottom row – ml/kg/hr. The first column shows treatment of crystalloid to colloid on a 1:1 basis. The second column shows treatment of crystalloid to colloid on a 3:1 basis. The third and fourth column are again 1:1 and 3:1 treatment, but instead of total amount administered look instead at fluid balance (amount administered minus urine output and blood loss). None of the regressions indicate a significant relationship between longer NPO time and higher fluid requirements by GDFT protocol

Figure 3 shows scatterplots of NPO time to fluid volume. Absolute adjusted and unadjusted R^2^ values are < 0.01 for each plot, indicating virtually non-existent correlations between uncorrected NPO time and fluid volumes measured by any of the 12 approaches in the figure.

## 5. Discussion

Despite lengthened NPO periods for PM surgeries, the GDFT algorithm did not result in an increased fluid administration rate for PM cases compared to AM cases. This finding agrees with suggestions from previous studies that the NPO period does not significantly impact required fluid delivery [1, 3, 5, 14]. Our study design adds to the growing evidence supporting withholding NPO period fluid replacement, while introducing reduced opportunity for inter-provider variability to influence fluid administration rates by allowing the GDFT algorithm to guide fluid delivery.

Our observation of greater epidural use, longer case duration, and greater use of laparoscopy in the AM group likely reflects two scenarios more likely to receive goal-directed fluid therapy: same-day surgery admit laparoscopic abdominal surgery with controlled ventilation and complex, and longer-duration, open abdominal surgeries with increased epidural usage that practically benefit from beginning earlier in the day. Therefore, complex surgery was overrepresented in the AM group as compared to the PM Group; however, our analysis did as much as possible to account for the types of differences in fluid requirement a more complex case may dictate. Specifically, removal of cases with > 500 ml of blood loss or those patients that received blood should have removed much of this variability, and correction for EBL, arterial line placement, and case duration in the models should have further corrected for this source of potential bias.

NPO time difference between AM and PM surgical case starts differed by a surprisingly small number of hours (less than 3), when we expected closer to 5. The small difference likely reflects both variation in how patients follow NPO guidelines, and the fact that the recorded NPO time remains subject to both recall and reporting bias. Moreover, the distribution of NPO times suggests bias in time entry by providers, which was part of the rationale for straight splitting of the cases into first-case starts and afternoon starts (as opposed to using raw NPO time as a covariate), because the NPO time entered may not have been completely reliable. We discuss this further in the limitations below.

AM starts actually received more fluid than PM starts when guided by GDFT. Some of those additional factors contributing to this finding may include patient pathology, condition, higher EBL, epidural vasoplegia, and greater insensible loss typical of more complex surgery as previously noted, though this result remains difficult to interpret in such a broad and multifactorial setting. Nevertheless, the variables affecting this previous result, namely, possible different ASA classifications among the AM vs. PM patient groups [4, 16, 17], the possible differential epidural placement for different cases [18, 19], the possible difference in the number of laparoscopic procedures in the AM vs PM groups [20, 21], different possible urine loss in cases of differing nature [22, 23], possible different patient demographics in weight [23–25] or in age [26–28], the different pragmatic scheduling need for surgical procedures of longer duration in the AM vs the PM groups [29–31], and the possible different hemodynamic heat rate parameter between surgical cases of different nature among the AM vs PM groups [21, 32, 33], have all been accounted for in the literature with evidence showing the clear benefit of following the GDFT algorithm. In some regards these limitations result from and are common to how operating rooms actually run. Because of (perhaps in spite of) this, our study may still answer the question of whether a patient presenting to surgery in the PM need different amounts of fluid than a patient presenting in the AM.

Thus, we believe there remains strong evidence to support a change in focus from “replacing NPO time deficits” to thinking more about patient and surgical case complexity. Obvious factors including presenting diagnosis, comorbidities, age, body habitus, expected blood loss, and case duration should be accounted for along with fluid responsiveness. Given the preponderance of variability in the literature with regards to the reliability of void urine output during surgery as a marker of volume status, void urine output in our study did indeed independently predict recommended fluid administration from the GDFT algorithm, in line with the earlier studies in literature that did suggest the predictable nature of void urine for fluid delivery [22, 23]. These data thus strongly support and importantly reinforce that a one-size fits all approach to fluid therapy does not benefit the surgical patient and should end where in use, shifting attention to the benefit of using the GDFT algorithm for resuscitation with our study’s supportive evidence of avoiding the NPO periods as a guide for excess fluid administration.

### 5.1. Limitations

The primary limitation of this study is the retrospective design. As with all retrospective studies, selection and information bias cannot be completely accounted for, and may confound the interpretation of the results. Missing or invalid data points in our database may have reduced the size and power of our study, but because the proportion of excluded and missing data was similar between the two groups, we believed the validity of the study remained intact. Additionally, excluding patients with EBL greater than 500 ml for reasons discussed above, while reducing variability, may have also introduced some selection bias. However, we used generalized linear models to help mitigate possible confounding factors and strengthen our results. The method of monitoring fluid responsiveness varied within our cohorts and we do not have specific records of which methods were used in which patients. There may be unaccounted bias in the approaches used between the AM and PM cohorts. Finally, the types of fluid used during resuscitation obviously differed from case to case and were not standardized. Despite this, there were not significant overall group differences, and we attempted to account for this in our results by creating multiple parallel analyses using different recommended replacement ratios from the literature. The consistent non-significant findings regardless of the approach used are reassuring that our findings are not approach-dependent.

A limitation of the NPO time comparison between groups, and indeed a limitation of the using the NPO time in general as a covariate in the models, was that the data entry in the EMR for the NPO period was not distributed as would be expected if precise times were being entered. Entries overwhelmingly fell at the 8 h, 10 h, and 12 h blocks, with the remaining periods being more evenly distributed as might be expected for “true” times (see figure below). In looking at the raw data, it appears that particular anesthesia providers defaulted, for example, to 8 h, or 10 h, and rarely entered ‘exact’ times. This was a key part of our rationale for simply dividing the cases into AM and PM groups since the raw NPO time was unreliable.

## 6. Conclusion

We set out to determine whether or not surgical case start time had any observable effect on intraoperative fluid requirements when guided by a GDFT protocol. Our conclusions, in agreement with prior work, suggest that NPO periods do not significantly influence a patient’s intraoperative fluid requirement and PM case starts do not require more fluids than AM case starts in any situation we could identify within our dataset. Future work could examine the repeatability of our findings in a multi-center and prospective study design.

## Supplementary information

**Additional file 1.** GDFT Protocol UCI 2019.pdf. Appendix: GDFT Protocol.

**Additional file 2.** GDFT Overview UCI 2019. Appendix: Overview document of our GDFT implementation.

## Data Availability

All data can be furnished upon reasonable request by email at the correspondence email address.
