# Supplementary material for: Lack of impact of nil-per-os (NPO) time on goal-directed fluid delivery in first case versus afternoon case starts: a retrospective cohort study
Source: BMC Anesthesiol. 2019 Oct 27;19:191. doi: 10.1186/s12871-019-0864-x (PMC6815464; doi:10.1186/s12871-019-0864-x)
Supplement: Supplementary file 1 — Additional file 1. GDFT Protocol UCI 2019.pdf. Appendix: GDFT Protocol. [file 12871_2019_864_MOESM1_ESM.pdf]

GDT Goal-Directed Therapy

UC Irvine Health School of Medicine | Department of Anesthesiology & Perioperative Care

Quality Improvement Protocol
Goal-Directed Fluid Therapy for Major Abdominal Surgery

Step 1: Monitoring Stroke Volume Variation (SVV)

- Fluid maintenance should be set to 3 ml/kg per hour; and controlled positive pressure ventilation at body weight with a **PEEP less than 10mmHG**. There should be no other fluids continuously infused.
- One should assess the stroke volume variation (SVV) **every 10 minutes**.
- Note that the monitor will alarm when the stroke volume variation has exceeded the 12 % threshold and if this occurs one should proceed through the protocol.
- SVV values should be consistent for **2 minutes** before following protocol treatment options.
- Note other factors can cause a change in stroke volume variation that does not relate to fluid responsiveness. This includes atrial fibrillation, non-controlled ventilation, and fluctuations in intra-abdominal pressure or thoracic pressure.
- If the number of cardiac arrhythmias are too frequent (such as Atrial Fibrillation) a Yellow Heart icon will appear next to SVV indicating that it can no longer be used to assess fluid responsiveness. A SV based protocol such as NICE/Kuper should be considered.

Step 2: SVV-Guided Fluid Intervention

- If the stroke volume variation is greater **than 12%** then one should give a **250ml bolus of colloid**. Colloids include: albumin or any hetastarch **(up to 1.5 L)**. Note that this protocol SHOULD NOT impact blood product administration in either transfusion thresholds or volume of products.
- After the colloid has been administered one should reassess the SVV again looking for the percent change **over 2 minutes**.
- If the value is now less than 12%, then this is showing that patients stroke volume optimization is improving. However, if the **SVV is between 8 to 12%** then one will need to further investigate if the patient may still benefit with additional colloids.
- To determine if the patient may still benefit from additional colloids, look for **a decrease in stroke volume (SV)** of greater than 10% over the past 5 minutes.
- The EV1000 clinical platform will calculate the percent change in SV automatically (bottom right hand corner).
- If this value shows a **> 10% decrease** over the past 5 minutes then one should **proceed with a colloid bolus**.
- If reassessed SVV is now less than 8% then one can confidently presume that stroke volume has been optimized and proceed to Step 3.

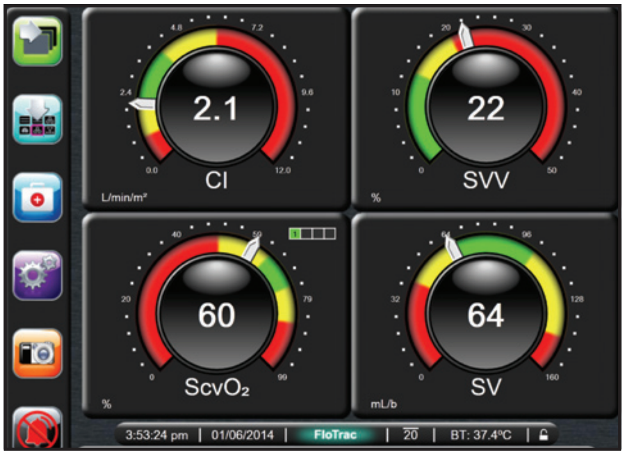

Figure 1 - Screenshot from the EV1000 monitor
Carry out the steps just described using this screen to identify the patients SVV, SV, and CI

Step 3: Assessment of BP

- If SVV and SV is in target range and BP is within 20% of target values then continue to monitor.
- If SVV is in target range but BP is below 20% of target values then assess the CI.
  - If CI is < 2.5 consider inotropic agent
  - If CI is > 2.5 consider vasoconstriction agent
- Even if the patient is on either inotropic or vasoconstriction agents one should continue to assess the percent change in SV every 10 minutes to determine if the patient is fluid responsive.

Goal-Directed Therapy

UC Irvine Health School of Medicine | Department of Anesthesiology & Perioperative Care

Quality Improvement Protocol
Goal-Directed Fluid Therapy for Major Abdominal Surgery

SV Optimization for Fluid Administration

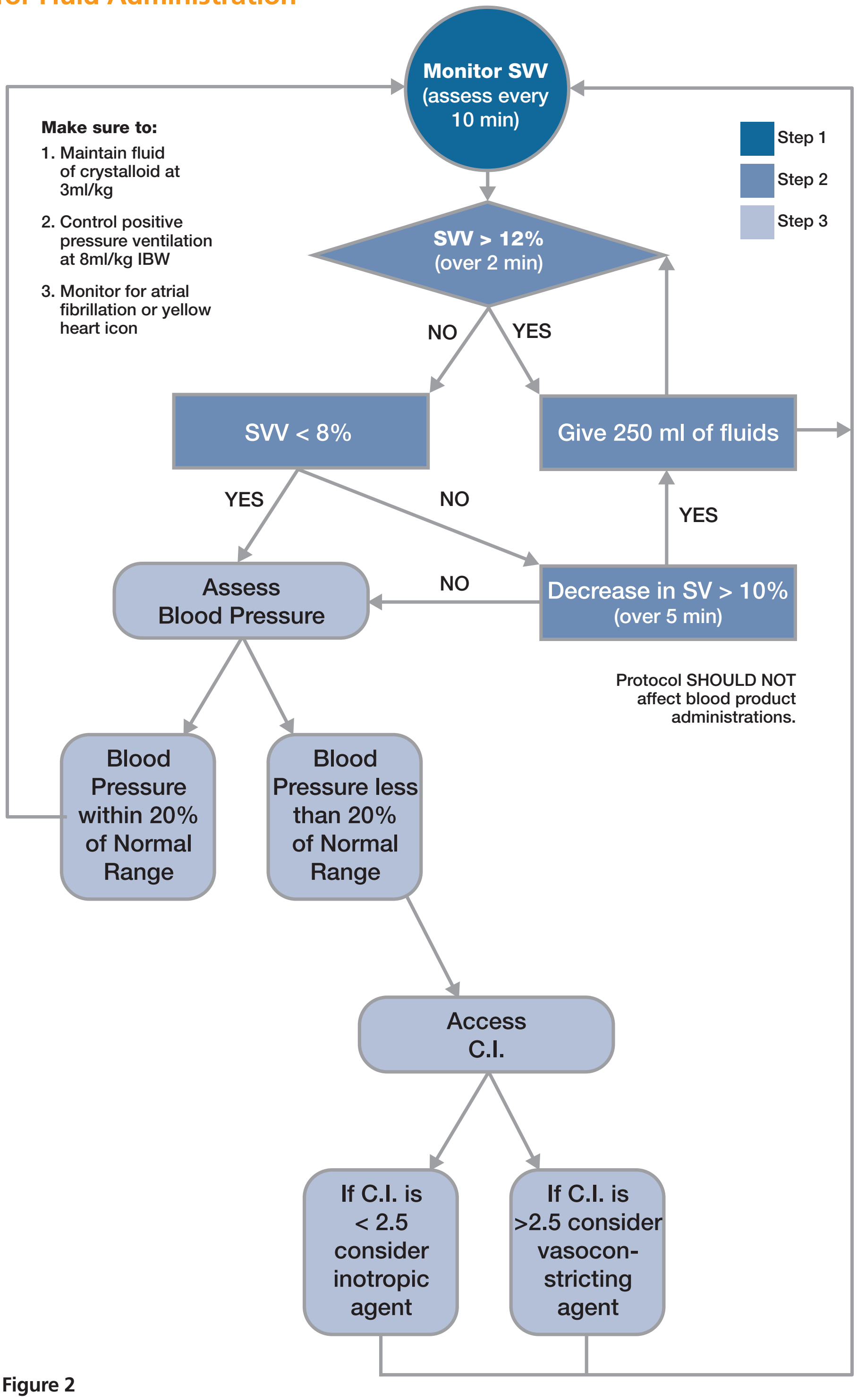

Figure 2
