# Supplementary material for: Lack of impact of nil-per-os (NPO) time on goal-directed fluid delivery in first case versus afternoon case starts: a retrospective cohort study
Source: BMC Anesthesiol. 2019 Oct 27;19:191. doi: 10.1186/s12871-019-0864-x (PMC6815464; doi:10.1186/s12871-019-0864-x)
Supplement: Supplementary file 2 — Additional file 2. GDFT Overview UCI 2019. Appendix: Overview document of our GDFT implementation. [file 12871_2019_864_MOESM2_ESM.pdf]

Goal-Directed Therapy

UC Irvine Health School of Medicine | Department of Anesthesiology & Perioperative Care

Quality Improvement Project Overview
Goal-Directed Fluid Therapy for Major Abdominal Surgery

Overview

- The QI project is based on the concept that either too little or too much fluid administration during the perioperative period can worsen organ function.
- Fixed volume strategies may be insufficient for perioperative fluid therapy.
- CVP, PAOP, and urine output are poor indicators of flow status.
- Because of the concerns of fixed fluid strategies, many anesthesiologists have proposed goal-directed fluid administration using dynamic (blood flow-related) hemodynamic variables such as stroke volume optimization.
- Recent technology utilizes analysis of the arterial waveform (pulse waveform contour analysis) and measures, continuously: cardiac output, stroke volume, PWCA, and stroke volume variation (SVV).
- Intraoperative goal-directed therapy in arterial pulse waveform contour analysis (PWCA) has been shown to improve post-operative morbidity.
- For this project at UCI the **Edwards EV1000** clinical platform will be utilized.
- This technology is based on the information that can be gleaned from analysis of the arterial waveform. Specifically the systolic upstroke (shown as point a on the diagram below) represents ventricular contractility. The diastolic downslope (point C) represents systemic vascular resistance and point D the area within the curve represents the stroke volume.

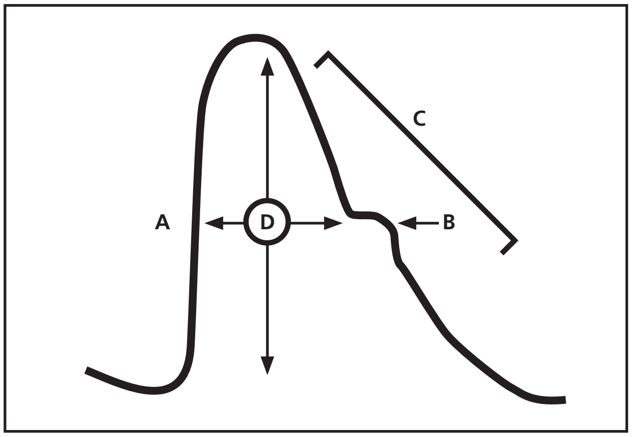

Figure 1

- It is important to always remember that a good arterial tracing is crucial for accurate information to be generated from these devices and one should always confirm proper leveling of the sensor to the phlebostatic axis as well as assure a proper square wave test is present.
- Knowing these flow-guided parameters of cardiac output, stroke volume and the variation in these parameters can help guide patient fluid management.
- A patient is fluid responsive when they have a >15% increase in CO in response to a fluid challenge (indicating that they are on the steep part of the Frank-Starling Curve).
- Stroke volume variation is a modality that can be used to identify patients who are on the steep portion of the Frank-Starling curve and are fluid responsive.
- The concept of SVV is based on the phenomena that positive pressure ventilation causes changes in venous return, which is accentuated in hypovolemic patients.
- During the inspiratory phase of positive pressure ventilation the increased intrathoracic pressure will cause the left side of the heart to initially receive additional volume from the pulmonary system. In addition the positive pressure will assist in LV contraction. Both of the these result in an increase in SV during inspiration of a positive pressure breath (definition of this phenomena being reversed pulsus paradoxus).
- On the right side of the heart the intrathoracic pressure increases from a positive pressure breath, which will increase right atrial pressure, causing venous return to decrease. This decreases right ventricular output, which then after two or three heart beats will manifest in a decrease in left ventricular output.
- Patients whom manifest greater change in SV as a result of positive pressure ventilation are more hypovolemic and therefore monitoring stroke volume variation has been demonstrated to accurately predict fluid responsiveness.
- Specifically, a large pulse pressure/stroke volume variation (> 12%) is indicative of hypovolemia and predictive of volume responsiveness.

Quality Improvement Project Overview
Goal-Directed Fluid Therapy for Major Abdominal Surgery

Heart-Lung Interactions with Positive Pressure Ventilation

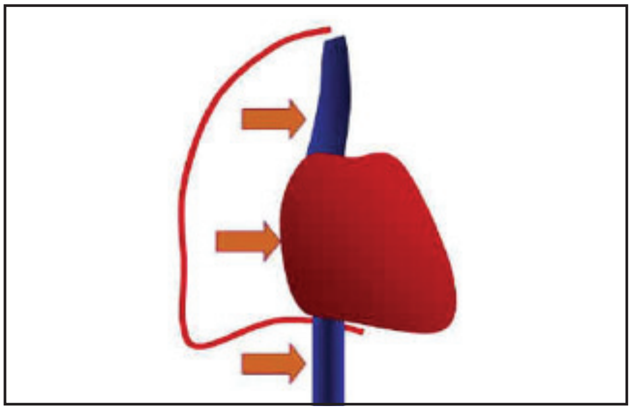

Figure 2 - Volume resuscitated patient

- Venous return does not fall during inspiration on SVV.
- Intrathoracic pressure is positive.
- Intra-abdominal pressure also rises.
- Pressure gradient between the abdomen and thorax is maintained.

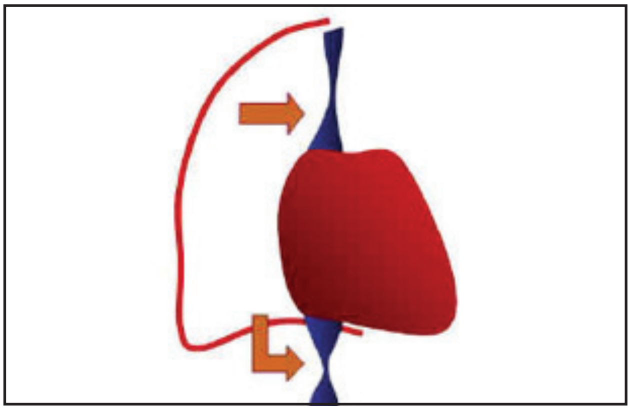

Figure 3 - Volume depleted patient on SVV

- Collapse of intra-abdominal veins and SVC occurs as a result of positive intrathoracic pressure.
- This results in a fall in venous return, RV stroke volume, LV preload, and cardiac output.

Requirements to use Stroke Volume Variation in determining Volume Responsiveness

1. Ability to generate intrathoracic pressure, closed chest conditions
2. Regular changes in intrathoracic pressure (controlled positive pressure ventilation at 8ml/kg of IBW)
3. Able to filter out arrhythmias - (Yellow Heart icon next to SVV on EV1000 = too many arrhythmias to filter (A-fib)
